# Evaluation of Coke Resistivity for the Manganese Alloy Market

**DOI:** 10.3390/ma15082897

**Published:** 2022-04-15

**Authors:** Jonathan Nhiwatiwa, Robert Cromarty

**Affiliations:** Department of Materials Science and Metallurgical Engineering, University of Pretoria, Pretoria 0002, South Africa; jonathannhiwatiwa@yahoo.co.uk

**Keywords:** coke resistivity, submerged arc furnace, single-source coal, four-point technique

## Abstract

The submerged arc furnace (SAF) has become the equipment of choice to produce manganese ferro-alloy. Furnace operators aim to reduce the cost of production by better understanding the role played by the various raw materials involved in the process. Coke is one of the key raw materials fed into the SAF; it plays three key roles in electric furnaces: as a reducing agent, as a source of carbon found in the alloy, and as a resistive element facilitating heat generation in the furnace. The heat generated plays two key functions in the furnace: ensuring both the metal and the slag have a sufficient low viscosity, and providing the heat required to support endothermic reactions. This study investigated the ambient-temperature and high-temperature resistivity characteristics of coke made from single-source coals. The measurement of coke resistivity was performed using the Kelvin (four-point) technique. The results showed a statistically significant difference in mean resistivity between cokes made from different coals. It was observed that coke resistivity generally decreased with increasing temperatures. Raman spectroscopy showed that the structural order of coke changes with increasing temperature.

## 1. Introduction

In the past, ferro-manganese was produced primarily through blast furnace processes. However, the submerged arc furnace (SAF) has gained prominence in recent times. The availability of relatively cheap electricity, the increased flexibility in operations, and the difficulty in sourcing the coals required to produce coke needed for good blast furnace operation resulted in a shift to the SAF. Both the blast furnace and the SAF use coke in their operations. Coke required for the BF has relatively stringent cold and hot strength requirements, while these requirements are relaxed for the SAF. Coke resistivity is not a factor in the operation of the BF, while it is important in the operation of the SAF. The literature on the coke quality requirements for the blast furnace is easily available [1,2]. However, there are fewer publications on the requirements for coke in the SAF. An advanced understanding of the coke quality requirements of the SAF would promote the efficient and cost-effective production of manganese alloy.

The SAF operates on the principle of resistive heating to generate heat required for the various reduction reactions and to promote adequate slag/metal separation. In the production of manganese alloy using the SAF furnace, a relatively high burden resistance is required to promote good heat generation and distribution within the furnace [3,4,5]. At furnace hearth temperatures, the solid burden consists mostly of solid coke and, thus, one of the key quality aspects of the coke used by the SAF should be its resistivity. Knowing the resistivity of the coke used is of crucial importance in the effective production of manganese alloy using the SAF.

Coke is a carbon source made from a fusible coal or coal blend that has passed through a semi-liquid phase to form a porous carbon material [6]. Parameters likely to affect coke resistivity include rank of the parent coal or coal blend, coke structural order, coke porosity, and coke ash content [7].

Several researchers have studied coke structural order and changes due to graphitization brought about by high temperature using Raman spectroscopy [8,9,10,11]. These authors concluded that the differences in coke structural order can be explained by the differences in Raman parameter characteristics.

The aim of this study was to investigate the existence of significant differences in coke material resistivity due to different parent coal properties such as carbon forms, porosity, and ash content. The study also aimed to establish correlations between coke resistivity and easily measured coke properties such as carbon forms, porosity, and ash content. It is anticipated that the knowledge gained would allow for optimization of the properties of coke intended for SAF production of manganese alloys.

## 2. Materials and Methods

Five coal types were provided by ArcelorMittal (Gauteng, South Africa). Three coals, from Australia and the USA, were low- to mid-volatile matter, one from Mozambique was a low volatile matter, and one from South African was a high-volatile semisoft coking coal.

The coal was brought in by rail and offloaded using a rail tipping station onto conveyor belts which conveyed it to the storage pads. An automatic belt sampler was used to collect increments at a time interval during the offloading to produce a 5 t composite sample of each coal type. The 5 t composite sample was further split to produce a 400 kg sample for coking in a pilot plant and a 50 kg sample for coal characterization. The 50 kg sample was then split further according to guidelines contained in ISO 13909 [12].

Procedures formulated from both American Society for Testing Materials (ASTM) and International Organization for Standardization (ISO) standards were used in the coal characterization test methodologies. Coal proximate analysis was carried out using a Leco thermogravimetric analyzer (TGA) model 601 (St. Joseph, MI, USA) that was calibrated with standards supplied by Leco International. Sulfur and ash chemistry tests were performed using the Leco tube furnace and X-ray spectrometer, respectively. All coal analyses were carried out by a commercial testing laboratory.

Table 1 gives results of the coal proximate and ash analysis of the coals. The imported coals, Coals A, B, C, and D, had relatively low volatile matter (range 20.3% to 29.6%) compared to the South African coal, Coal E, with an average volatile matter of 37.6%; these results are consistent with expectations from the respective sources.

Coal petrography was carried out on the coal samples using a Zeiss Axio Imager M2M (Carl Zeiss Microscopy LLC, White Plains, NY, USA) Wat a magnification of 50 under oil immersion. The sample was first crushed to less than 850 μm and then mounted in epoxy resin before polishing using standard ASTM guidelines [13]. The preparation and analysis procedures were carried out by a Society for Organic Petrology-accredited coal petrographer. The coal petrography results are shown in Table 2.

**Figure 1:**
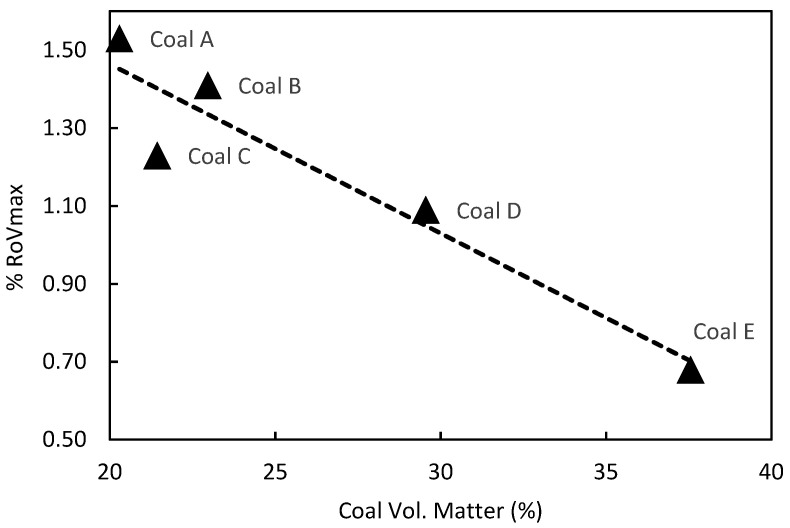
Parent coal rank, expressed as %RoVmax, as a function of volatile matter.

The petrography results were consistent with the proximate analysis results in Table 1. In general, volatile matter content decreases with increasing rank as measured by the reflectance of vitrinite or reflectance of reactive macerals (RoVmax or RoRmax). This is illustrated in Figure 1 showing coal rank, expressed as RoVmax, as a function of percent volatile matter in the coal.

The pilot coke oven has comparable width dimensions to a typical industrial coke oven and is believed to produce coke that is comparable in properties to that produced by industrial coke ovens [6]. Using pilot coke ovens in research has the added advantage that they can produce relatively small amounts of coke from single sources without the risk of damage to the integrity of the coke oven structure. The 400 kg coal sample for each coal source collected was reduced in particle size to about 80% (3.35 mm) using a hammer mill as per the requirement when producing industrial coke with the top charging method. The oven temperature control set points were kept similar for the five coals under study. Table 3 gives the coking conditions achieved for each coal during the coking process.

About 250 kg of coke was produced by the pilot oven for each coal type. The coke was split using standard preparation methods to produce a 50 kg sample for coke characterization. The remaining coke was used to prepare test pieces for coke resistivity tests. Table 4 gives the coke characterization results for the five coke types produced. As with the coal, standard ISO and ASTM methods were used for the analysis [14,15,16,17,18].

Coke porosity was measured using an Autopore IV 9500 V1.09 from Micromeritics Instrument Corp. (Norcross, GA, USA). Test pieces were sent to Wuhan University of Science and Technology, and the porosity analysis was carried out there.

**Figure 2:**
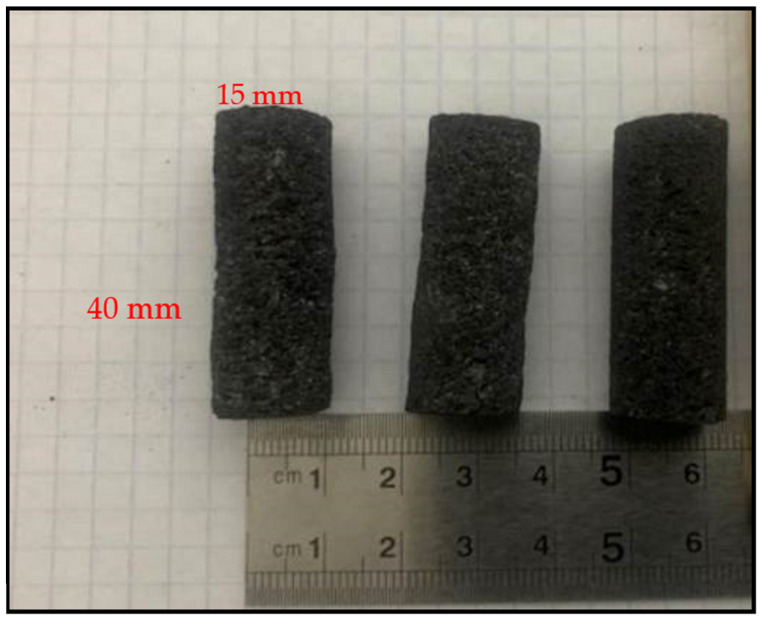
Coke resistivity test samples.

Coke pieces from each coke source were machined using a diamond-tipped core drill to produce cylindrical coke pieces with a diameter of about 15 mm and length of 40 mm as shown in Figure 2. Coke resistivity measurements were performed at ambient temperatures and at temperatures up to 1600 °C.

**Figure 3:**
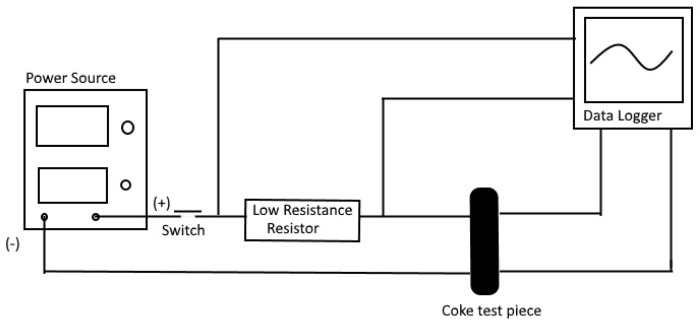
Schematic representation of the setup to measure ambient temperature resistivity.

A four-point method, as shown in Figure 3, was used to measure the resistance of the coke pieces in ambient temperature.

Ten pieces of each coke type were measured. For each sample, the resistivity was calculated using the distance between the voltage sensor contacts and the cross-sectional area of the sample. Sample diameter was measured at three points—two ends and center.

**Figure 4:**
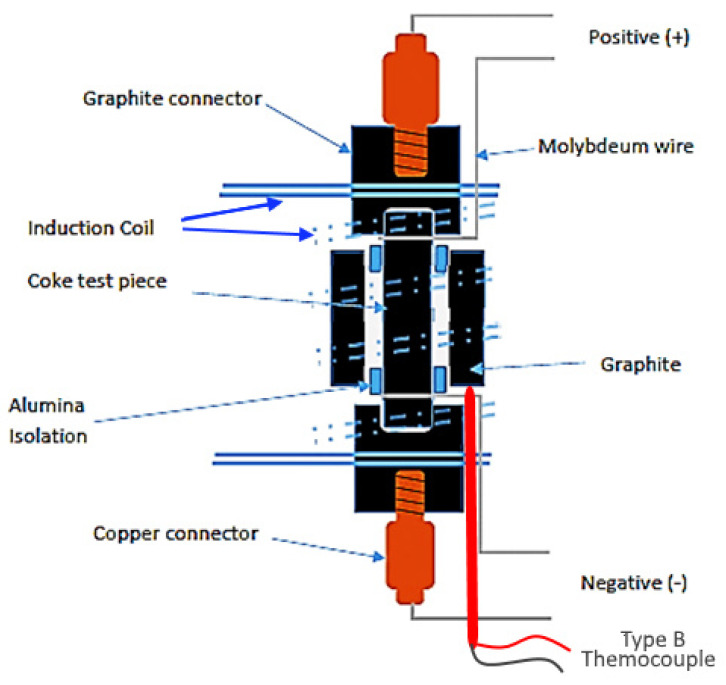
Schematic representation of the arrangement to measure high-temperature resistivity.

Due to the difficulty faced in obtaining conductive material able to withstand temperatures of up to 1600 °C in the presence of carbon without either melting or forming nonconductive carbides, molybdenum wire was used to form connections in the areas where temperatures reached 1600 °C or higher. The whole assembly was insulated and placed in an induction coil arrangement powered by a Ambrell Ekoheat 15 kW induction power supply unit (Rochester, NY, USA). Figure 4 shows a schematic diagram of the setup used to measure coke resistivity at high temperature.

The rest of the electrical circuit consisted of a power source and data logger with a similar circuit to that used for the ambient temperature coke resistance measurements in Figure 3. Coke resistance readings were collected while the temperature was incrementally increased by increasing the applied power. As for the ambient temperature measurements, the sample resistance was converted to sample resistivity.

In this research, a microscope-mounted Horiba Jobin Yvon TX6400 Raman spectrometer (Edison, NJ, USA) with a nitrogen-cooled detector was used to analyze the coke samples before and after heat treatment. Origin software was used to deconvolute the Raman spectra. A Gaussian function was fitted on the four common carbonaceous material bands found; specifically, the D1, D2, D3, and G bands and the respective peak parameters were recorded. Once the curves are deconvoluted, some characteristic parameters of each of the bands can be determined. These Raman parameters can be used to describe the characteristics of the material being studied. The common Raman parameters are peak position, peak intensity, and the full width at half maximum (FWHM). The ratios of these parameters can be used to describe differences in materials as well [8,9,10,11].

Analysis of variance (ANOVA) was used to test if there was a statistically significant difference in ambient temperature resistivity.

To investigate the relationship that exists between coke resistivity measurements and other coke properties, correlational studies and multivariate techniques were used. The ambient resistivity dataset was larger than the high-temperature resistivity dataset; hence, only the ambient temperature dataset was used for correlational analysis.

## 3. Results

### 3.1. Ambient-Temperature Coke Resistivity Tests

**Figure 5:**
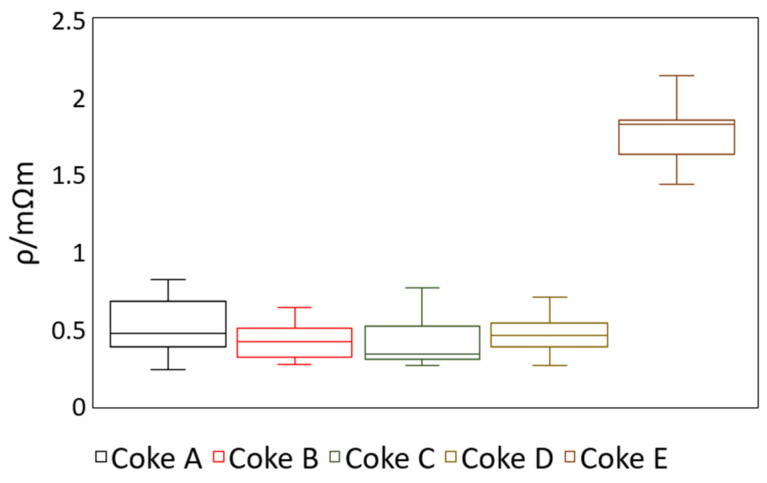
Room-temperature coke resistivity box-and-whisker plot. Sample size: 10 samples for each coke type.

Figure 5 shows the box-and-whisker plots of the ambient-temperature coke resistivity results.

Cokes A, B, C, and D had average coke resistivity measurements in the range 0.41 mΩ·m to 0.52 mΩ·m. Coke resistivity readings of Coke E were in the range from 1.44 mΩ·m to 2.14 mΩ·m; these were significantly higher than the resistivity readings of the other coke types.

From Table 1 and Figure 1, it can be seen that Coke E was produced from a low-rank coal. From Table 5, it can be seen that Coke E had a much higher isotropic carbon content than the other four coke types.

The probability that the mean coke resistivities of all the coke types were the same was 1.26 × 10^−22^. This probability is much smaller than the test significance level of 0.05. This means that the difference of the means amongst the coke types was significant, i.e., at least one of the mean coke resistivity results was significantly different from the others in the data analyzed. This result does not tell us how many of the possible 10-paired combinations were significantly different from each other. To get more information about the significant ANOVA result, a post hoc test was conducted.

There was a statistically significant difference between the mean resistivity of Coke E and the other four coke types when using both the Bonferroni and the Holm–Bonferroni methods. None of the other coke types showed a statistically significant difference in mean resistivity. This can also be seen clearly from Figure 5.

### 3.2. Effect of Temperature on Coke Resistivity

**Figure 6:**
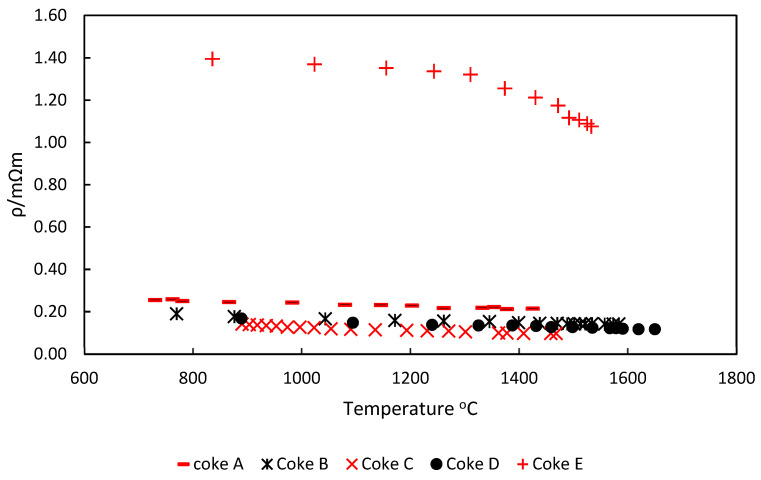
Change in coke resistivity with increasing temperature.

In Figure 6, the change in coke resistivity with increasing temperature is shown for each of the coke types. The results showed a similar trend to the coke resistivity measurements obtained at room temperature. For the same conditions, Coke E had a resistivity significantly higher than Cokes A, B, C, and D. This corresponds to the room-temperature resistivity measurements.

In Figure 6, it can be seen that the resistivity of Cokes A, B, C, and D decreased as the temperature increased. Other authors have also noted a similar result and have attributed this to a possible change in the microstructure when coke is heated to temperatures beyond the coking temperatures [7,19,20].

With respect to the other coke types, Coke E showed a decrease in resistivity as the temperature increased. The resistivity of Coke E was reduced more significantly beyond temperatures of about 1300 °C. However, a significant difference was still present between Coke E and the other coke types even at temperatures close to 1600 °C.

Krogerus, Lintumaa, and Jokinen [7], while investigating the conductivity of metallurgical coke, char, gas coke, and several other mixtures, concluded that the resistivity of these carbonaceous materials generally decreased as the temperature increased toward 1600 °C. This conclusion is consistent with observations in the current work for all coke types. Eidem [19] and Nurmukhanbetov, Prokopyev, and Privalov [20] came to similar conclusions.

The number of tests carried out at high temperatures was limited by the difficultly encountered in taking such measurements continuously without significant damage to the test setup. Repeatability of the results could not be adequately tested at high temperature as was the case at ambient temperature.

### 3.3. Raman Spectroscopy

The results show that changes in structure occurred when coke was heated to beyond the initial coking temperatures.

Four distinct Raman spectra peaks were identified for the five coke types under study. These peaks were at 1580 cm^−1^ (G band), 1350 cm^−1^ (D1 band), 1620 cm^−1^ (D2 band), and 1500 cm^−1^ (D3 band).

For all coke types, a prominent G band was observed. The second most significant band present was the D1 peak. This band was observed for all coke types included in the study before and after heat treatment of the samples.

Where the D2 band peak was visible, it appeared as a shoulder on the G band peak. In the current study, the D2 peak of the coke before heating was not clearly identifiable due to the broad G peak. In some cases, the analysis software could not adequately separate the G and D2 bands due to their proximity. This can be seen by the tendency of distortion to the G band toward high Raman shift positions where the analysis software failed to detect the D2 band. It is the authors’ understanding that, in such cases, the D2 band is likely present although the curve deconvolution software failed to detect it. In some coke types, the D3 band was observed with a position between the D1 and G bands.

**Figure 7:**
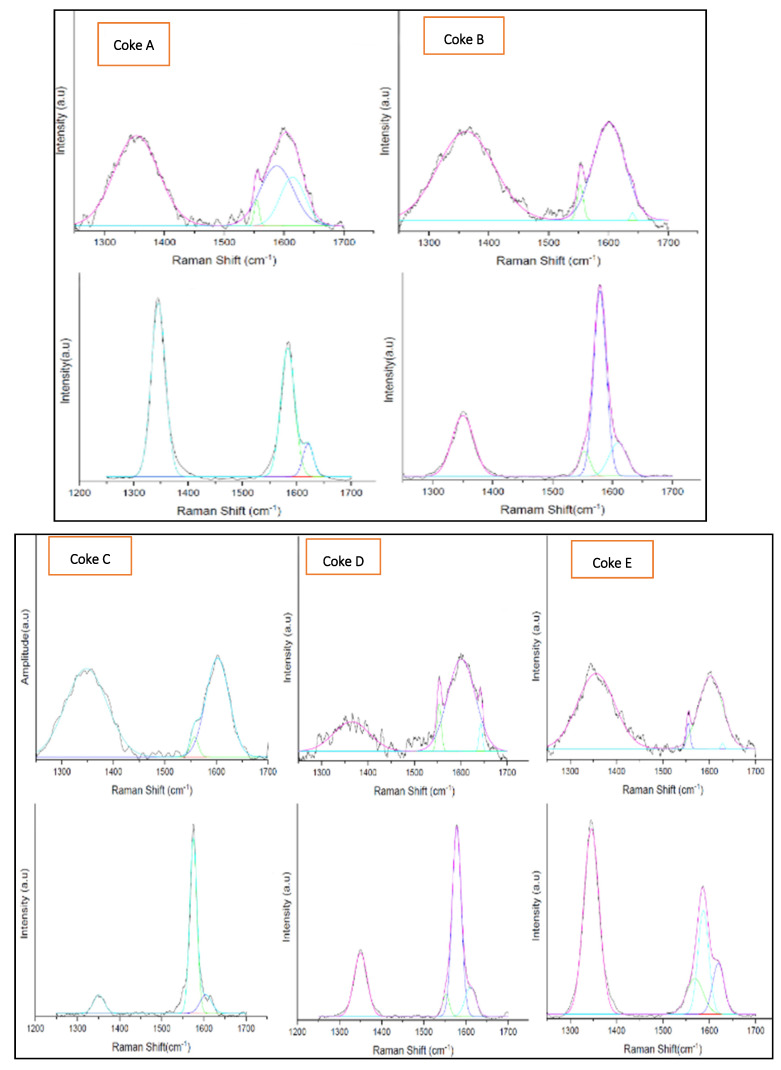
Comparison of the Raman spectra of all five coke types before and after heating to temperature up to 1600 °C. Top row: before heating; bottom row: after heating. Continous curve is the original curve and the rest of the curves are deconvoluted curves of the various characteristics curves.

Figure 7 shows the Raman peaks of coke before and after heating to a temperature of 1600 °C.

**Figure 8:**
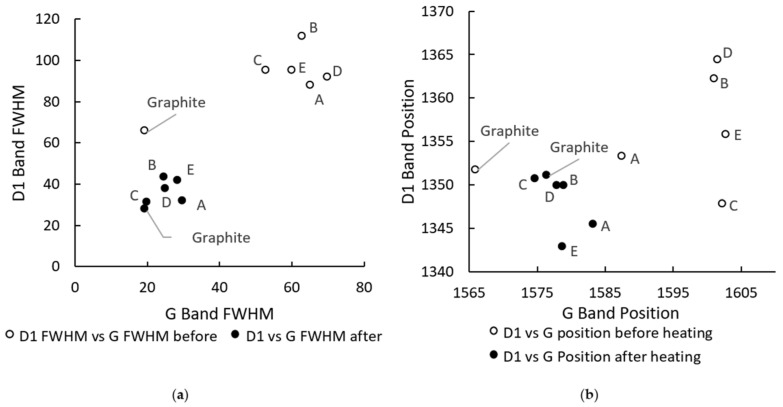
Raman parameter ratios for coke before and after heating to 1600 °C: (**a**) change in the coke FWHM of the D1 band vs. that of the G band before and after heating; (**b**) change in G band position vs. D1 position due to heating.

Figure 8a,b show the effect of heat on the D1 against G FWHM ratio and the D1 against G position ratio.

In both cases, the magnitude and dispersion of the ratios appeared to reduce after heat treatment. This suggests an increase in graphitization of all coke types during heating.

The coke resistivity and Raman results support the observation that structural order increases with the heat treatment of coke. This observation agrees with conclusions by other authors in earlier work [8,9,11,21].

No clear correlation could be found between the change in coke resistivity when heating and the change in Raman parameters.

### 3.4. Correlation Results and Discussion

To understand the relationship between resistivity and measured coal and coke properties, correlational studies were carried out using the SPSS statistical software. The initial study involved a bivariate correlational analysis of the ambient-temperature coke resistivity results with coke porosity, coke ash content, percent isotropic carbon, and rank.

Coke porosity shows only moderate correlation with coke resistivity and moderate to weak correlation with the other properties. This result is likely because porosity is largely influenced by the method of coke making used, in this study all the coke was top charged without any stamping.

To further study the relationships among coke resistivity, percent isotropic carbon, porosity, and ash content, partial correlations were used. Table 6 shows the results of the partial correlation between coke resistivity and percent isotropic carbon while controlling for ash content and porosity.

In Table 6, the partial correlation of the percent isotropic carbon remained strong compared to the bivariate correlation (difference < 0.1) even after controlling for both porosity and ash content. This means there was an almost direct relationship between the percentage isotropic carbon and coke resistivity.

Table 7 shows the partial correlation of ash content compared to coke resistivity when controlling for the percent isotropic carbon and porosity.

Table 8 also shows the partial correlation of porosity compared to coke resistivity while controlling for the other two parameters.

In all tables, the bivariate coefficient was lower than the partial correlation coefficient. This means that the percent isotropic carbon, ash content, and porosity were independent variables related to coke resistivity.

Multiple correlational studies were carried out on the ambient-temperature coke resistivity data with percent isotropic carbon, ash, and porosity as the independent variables.

The results were used to model the coke resistivity given the percent isotropic carbon, ash content, and porosity and compared to measured values using Equation (1).

ρ = 0.164 + 0.036 × ash − 0.05 × porosity + 0.015 × isotropic carbon(1)

where ash is the mass percentage ash, porosity is the volume percentage porosity, and isotropic carbon is the percentage isotropic carbon in the coke. Table 9 shows a comparison of the average coke resistivity values with those calculated using the model in Equation (1).

## 4. Discussion

Coke resistivity was measured using the four-point measurement technique at ambient temperature and upon increasing temperature to beyond 1600 °C. Statistical analysis using the two-tailed single-factor ANOVA test yielded no significant difference in mean resistivity amongst Cokes A, B, C, and D at a 95% confidence interval. However, Coke E showed a significantly different mean coke resistivity, at ambient temperature, when compared to the other four coke types using the same method. This result was consistent with work by other authors on similar carbonaceous material [20].

The study of the effect of temperature on the different coke types was carried out using a four-point measurement assembly, but the arrangement was mounted inside an induction furnace. By studying the trend of coke resistivity results produced, it was observed that, for Cokes A, B, C, and D, the resistivity tended to reduce as the temperature increased toward 1600 °C. This observation is consistent with similar work carried out under similar conditions.

A plot of the D1/G FWHM ratio and the D1/G position ratio showed the changes in structure of coke after heating to temperatures beyond the coking temperatures for all coke types. This conclusion agrees with the results obtained by other authors [10,11,22].

Amongst the coke properties investigated, coke optical texture measured by the percent isotropic carbon, coke ash content, and coke porosity were seen to show a notable correlation with coke resistivity. Coke optical texture as measured by the percent isotropic carbon content showed a strong significant bivariate correlation coefficient. The other two coke properties showed strong and moderate bivariate correlation coefficients.

Partial correlational studies indicated an almost direct correlation between resistivity and the porosity, optical texture, and ash content of coke.

Linear multiple regression was used to model coke resistivity as a function of the coke optical texture, ash content, and porosity. Comparing the model predictions with the measured average resistivity data produced a good fit. It can, therefore, be concluded that coke resistivity can be predicted from these coke parameters.

In summary, the resistivity of coke is influenced by the parent coal used if the parent coal properties are significantly different. As the coke in a smelter is subjected to increasing temperatures, the respective coke resistivity tends to converge to similar values as coke graphitization occurs.

## Figures and Tables

**Table 1:** Proximate analysis and coal ash analysis of the parent coals (% by mass).

Coal Designation	A	B	C	D	E
Proximate analysis (dry basis)			-	-	-
Ash	12.1	9.7	9.5	9.4	10.2
Volatile matter	20.3	23.0	21.4	29.6	37.6
Fixed carbon	67.6	67.3	69.1	61.0	52.2
Ash chemistry	-	-	-	-	-
SiO_2_	6.172	4.966	6.015	5.017	6.181
CaO	0.280	0.234	0.054	0.226	0.150
MgO	0.057	0.009	0.008	0.066	0.009
Al_2_O_3_	2.064	3.068	2.195	2.186	1.618
Total Fe	0.440	1.901	2.701	4.656	3.965
MnO	0.005	0.021	0.009	0.038	0.029
TiO_2_	0.447	0.242	0.220	0.205	0.338
P_2_O_5_	0.195	0.169	0.039	0.106	0.014
P	0.085	0.074	0.017	0.046	0.006
K_2_O	0.177	0.103	0.078	0.195	0.089
Na_2_O	0.060	0.097	0084	0.121	0.091
Basicity index *	0.123	0.292	0.356	0.731	0.552

* The basicity index is the ratio of basic to acidic oxides in coke or coal.

**Table 2:** Coal petrography results.

Coal Type	A	B	C	D	E
Total vitrinite (%)	78.60	80.30	57.60	80.20	88.70
Total reactives (%)	78.60	81.00	71.30	85.40	90.70
Total inerts (%)	21.40	19.00	28.70	14.60	9.30
% RoVmax	1.53	1.41	1.23	1.09	0.68
% RoV random	1.43	1.32	1.15	1.02	0.64
% RoRmax	1.53	1.41	1.28	1.10	0.68

**Table 3:** Pilot oven conditions.

Coal Type	Coal A	Coal B	Coal C	Coal D	Coal E
Plastic zone (hours to 474 °C)	13.75	13.35	13.50	11.40	13.15
Coke rate (hours to 900 °C)	15.20	15.25	15.60	14.85	14.80
Coking rate, mm/h	31.12	31.02	30.32	31.85	31.96
Total coking cycle, h	17.05	17.05	17.40	16.60	16.40

**Table 4:** Proximate and ash chemical analysis of coke produced from the pilot ovens (% by mass).

Coke Type	A	B	C	D	E
Proximate analysis (dry basis)	-	-	-	-	-
Ash	13.3	12.0	13.0	12.5	14.0
Volatiles	0.8	0.8	0.9	0.9	1.0
Fixed carbon	85.9	87.2	86.1	86.6	85.0
Ash chemistry	-	-	-	-	-
SiO_2_	6.62	4.88	7.52	5.39	7.81
CaO	0.34	0.29	0.11	0.22	0.16
MgO	0.16	0.04	0.04	0.08	0.04
Al_2_O_3_	1.92	1.83	2.11	1.91	1.03
Total Fe	1.43	0.71	2.75	0.69	0.71
MnO	0.01	0.01	0.01	0.01	0.02
TiO_2_	0.54	0.30	0.28	0.32	0.50
P_2_O_5_	0.14	0.14	0.02	0.08	0.02
P	0.059	0.060	0.010	0.037	0.010
K_2_O	0.18	0.11	0.10	0.21	0.12
Na_2_O	0.08	0.10	0.11	0.09	0.08
Basicity index	0.257	0.187	0.323	0.176	0.125

**Table 5:** Coke carbon forms.

Coke Type	A	B	C	D	E
Isotropic (%)	3.9	2.6	2.7	0.4	90.0
Incipient (%)	2.0	0.0	3.3	1.2	6.0
Circular anisotropic (%)	90.6	87.2	93.0	98.4	4.0
Lenticular anisotropic (%)	3.5	7.9	1.0	0.0	0.0
Robin anisotropic (%)	0.0	2.3	0.0	0.0	0.0

**Table 6:** Correlation matrix of percentage isotropic carbon and coke resistivity while controlling for ash content and porosity.

Control Variables			Resistivity	Isotropic Carbon	Porosity	Ash
-none-	Resistivity	Correlation	1.000	0.994	0.592	0.805
	Isotropic carbon	Correlation	0.994	1.000	0.499	0.781
	Porosity	Correlation	0.592	0.499	1.000	0.549
	Ash	Correlation	0.805	0.781	0.549	1.000
Porosity and ash	Resistivity	Correlation	1.000	1.000	-	-
	Isotropic carbon	Correlation	1.000	1.000	-	-

**Table 7:** Correlation matrix of coke resistivity and coke ash content while controlling for both precent Isotropic carbon content and porosity.

Control Variables			Resistivity	Ash	Porosity	Isotropic Carbon
-none-	Resistivity	Correlation	1.000	0.805	0.592	0.994
	Ash	Correlation	0.805	1.000	0.549	0.781
	Porosity	Correlation	0.592	0.549	1.000	0.499
	Isotropic carbon	Correlation	0.994	0.781	0.499	1.000
Porosity and isotropic carbon	Resistivity	Correlation	1.000	0.985	-	-
	Ash	Correlation	0.985	1.000	-	-

**Table 8:** Correlation matrix of coke resistivity and coke porosity while controlling for both precent isotropic carbon content and ash content.

Control Variables			Resistivity	Porosity	Ash	Isotropic Carbon
-none-	Resistivity	Correlation	1.000	0.592	0.805	0.994
	Porosity	Correlation	0.592	1.000	0.549	0.499
	Ash	Correlation	0.805	0.549	1.000	0.781
	Isotropic carbon	Correlation	0.994	0.499	0.781	1.000
Ash and isotropic carbon	Resistivity	Correlation	1.000	1.000		
	Porosity	Correlation	1.000	1.000		

**Table 9:** Correlation matrix of coke resistivity and coke porosity while controlling for both precent isotropic carbon content and ash content.

Coke Type	Predicted Resistivity	Measured Resistivity	Percent Residuals
A	0.4865	0.5191	6%
B	0.4304	0.4293	0%
C	0.4722	0.4143	−14%
D	0.4406	0.4667	6%
E	1.7777	1.7780	0%

## Data Availability

The data presented in this study are available on request from the corresponding author.
